# Salophen

**Published:** 1894-01-20

**Authors:** 


### SALOPHEN.

Of the many salicylic compounds that have been brought forward salophen is probably one of the most valuable, and it has quickly received the support of distinguished clinical physicians, such as Gerhardt[1]. It consists chemically[2] of 50·9 per cent. of salicylic acid, and 49·1 per cent. of acetylparamidophenol, so that it may be regarded either as salicylate of acetylparamidophenolether or acetylparamidosaiol. It is a white crystalline powder, almost insoluble in cold water, slightly soluble in hot water, easily soluble in alcohol, ether, and caustic alkalies. It has no smell, and is tasteless. On account of its insolubility it is best given in cachets. The usual dose for an adult is about fifteen grains five or six times a day.

Salophen is split up in the intestine by the panerectic juice and the succus entericus into salicylic acid and acetylparamidophenol. This does not happen if large doses are given, for then it may be excreted unchanged in the urine. One very peculiar effect of it is that crystalline deposits are met with upon the skin[3] of those patients who are taking it. Many observers have described this, but the best account is by Dr. Drasche, who says that when the crystals are about to appear there is often profuse perspiration, and after that has ceased and evaporated a number of small graceful-pointed crystals are seen, which glimmer and flicker until some parts of the skin appear to be covered with diamond dust, especially the face, neck, breast, and sides of the limb. A white asbestos-like material may also be seen in the folds of the joints. The chemical nature of the crystals has not yet been made out, but they appear like crystals of salophen. Many observers have given salophen to patients suffering from acute rheumatism, and all are agreed that it lessens the pain and the fever quite as efficaciously as other salicylates, but that it does not diminish the liability to rheumatic endocarditis. The advantages claimed for it are, firstly, its tastelessness; secondly, that it does not produce the headache and tinnitus so common after sodium salicylate; and, thirdly, that it does not derange digestion. One of Hardenbergh's patients, a boy of sixteen, had two hundred and seventy grains in thirty-six hours without any ill effects. Like other salicylates, it is not of much use in chronic rheumatic arthritis. It is an excellent analgesic, and has been used in smaller doses in neuralgia, migraine, and headaches, and, if anything, is more efficacious than other salicylates.

[1] Inaug. Dissert., Jena, 1893. [2] Nouv. Rém., Sept. 30, 1823. [3] Deutsche Wiener Medizinische Wochenschrift, No. 29, 1892.

